# Cerium Oxide Nanoparticles Regulate Insulin Sensitivity and Oxidative Markers in 3T3-L1 Adipocytes and C2C12 Myotubes

**DOI:** 10.1155/2019/2695289

**Published:** 2019-02-04

**Authors:** Amaya Lopez-Pascual, Andoni Urrutia-Sarratea, Silvia Lorente-Cebrián, J. Alfredo Martinez, Pedro González-Muniesa

**Affiliations:** ^1^University of Navarra, Department of Nutrition, Food Science and Physiology, School of Pharmacy and Nutrition, Pamplona, Spain; ^2^University of Navarra, Centre for Nutrition Research, School of Pharmacy and Nutrition, Pamplona, Spain; ^3^IdiSNA Navarra's Health Research Institute, Pamplona, Spain; ^4^CIBERobn Physiopathology of Obesity and Nutrition, Centre of Biomedical Research Network, ISCIII, Madrid, Spain

## Abstract

Insulin resistance is associated with oxidative stress, mitochondrial dysfunction, and a chronic low-grade inflammatory status. In this sense, cerium oxide nanoparticles (CeO_2_ NPs) are promising nanomaterials with antioxidant and anti-inflammatory properties. Thus, we aimed to evaluate the effect of CeO_2_ NPs in mouse 3T3-L1 adipocytes, RAW 264.7 macrophages, and C2C12 myotubes under control or proinflammatory conditions. Macrophages were treated with LPS, and both adipocytes and myotubes with conditioned medium (25% LPS-activated macrophages medium) to promote inflammation. CeO_2_ NPs showed a mean size of ≤25.3 nm (96.7%) and a zeta potential of 30.57 ± 0.58 mV, suitable for cell internalization. CeO_2_ NPs reduced extracellular reactive oxygen species (ROS) in adipocytes with inflammation while increased in myotubes with control medium. The CeO_2_ NPs increased mitochondrial content was observed in adipocytes under proinflammatory conditions. Furthermore, the expression of *Adipoq* and *Il10* increased in adipocytes treated with CeO_2_ NPs. In myotubes, both *Il1b* and *Adipoq* were downregulated while *Irs1* was upregulated. Overall, our results suggest that CeO_2_ NPs could potentially have an insulin-sensitizing effect specifically on adipose tissue and skeletal muscle. However, further research is needed to confirm these findings.

## 1. Introduction

The metabolic syndrome is a complex interplay of comorbidities including central adiposity, dyslipidemia, hyperglycemia, and hypertension [1]. Over the last decades, this clustering of factors has been widely implicated in the pathogenesis of type 2 diabetes and cardiovascular disease [2, 3]. In the normal course of metabolism, the pancreatic *β*-cells release insulin which stimulates glucose, amino acid, and fatty acid uptake. However, when insulin resistance is present, as often happens in obese subjects, *β*-cells increase insulin secretion to maintain normal glucose tolerance [4]. Concerning insulin signaling, the phosphorylation of insulin substrate receptor 1 and 2 (IRS-1 and IRS-2) is a key cellular response for glucose uptake [5, 6]. Insulin resistance is related to many physiopathological features of metabolic syndrome such as the oxidative stress, mitochondrial dysfunction, and a chronic low-grade inflammatory status [5–8].

In this context, type 2 diabetes is a major public health problem, which has been extensively studied for prevention and therapy development [3], as the complex pathophysiology and the heterogeneous drug responses hamper the proper treatment of the disease [4, 9, 10]. New therapeutic approaches should identify additional targets [11], offering a more directed and therefore effective treatment for type 2 diabetes [6]. As novel strategies, antioxidant treatment has been proposed to combat oxidative stress in diabetic patients [5] as well as anti-inflammatory approaches to immunomodulate towards a more balanced insulin response [12]. In this sense, nanomedicine is being used in noninvasive approaches to treat metabolic-related diseases as type 2 diabetes [9]. The administration of nanostructured particles has shown a therapeutic potential due to a better distribution and cellular uptake than other drugs, as well as the transexcitation reactions that make them able to take part in redox reactions [13–15]. The cerium oxide nanoparticles (CeO_2_ NPs) are one of the most promising nanomaterials for antioxidant and anti-inflammatory pharmacological applications [13, 16, 17]. Hence, CeO_2_ NPs have been proposed for diverse biological purposes such as therapy for neurodegenerative disorders, oxidative stress-related diseases, diabetes, chronic inflammation, and cancer among others [13, 16, 18]. Moreover, cerium exists in two oxidative states: Ce^+3^ and Ce^+4^ [16]. The therapeutic benefit is attributed to its ability to mimic superoxide dismutase, behaving as efficient reactive oxygen species (ROS) scavengers (Ce^+3^ to Ce^+4^) and changing the oxidation state to mimic catalase activity that reduces hydrogen peroxide releasing protons and O_2_ (Ce^+4^ to the initial Ce^+3^). Therefore, this self-regenerative property renders the nanoparticles a very valuable tool for pharmacological treatment of oxidative-related disorders [13, 16]. Previous studies have evidenced useful properties of CeO_2_ NPs related to redox status modulation in many conditions such as macular degeneration [19], lung damage [20], liver toxicity [21], cardiac dysfunction [22], smoke-related cardiomyopathy [23], adipogenesis [24], and weight-gain reduction [25]. On the other hand, some authors described DNA damage and inflammation in the lung, heart, liver, kidney, spleen, and brain [26], inability to counteract monocyte inflammation [27], lung-cell apoptosis [28], and monocyte cell death through apoptosis and autophagy [29]. Consequently, the hypothesis of this study was that a treatment with nanoparticles could potentially attenuate type 2 diabetes features and metabolic syndrome markers in 3T3-L1 adipocytes and C2C12 myotubes. As aforementioned, the literature gives insight into the specific cell-type effect of this potential treatment. Thus, the objective of the present study was to evaluate the effect of CeO_2_ NPs on markers of oxidative stress, mitochondrial dysfunction, and inflammation in mouse adipocyte, macrophage, and myotube cell cultures under control or proinflammatory conditions.

## 2. Material and Methods

### 2.1. Cell Cultures

The cell lines were obtained from the American Type Culture Collection (ATCC®) and cultured according to the accompanying specifications. Concretely, mouse 3T3-L1 preadipocytes, C2C12 myoblasts, and RAW 264.7 macrophages (ATCC® CL-173™, CRL-1772™ and TIB-71™, respectively) were cultured in growth medium composed by DMEM (Gibco, NZ) with 25 mM glucose and 100 U/ml penicillin-streptomycin (Invitrogen, NZ), supplemented with 10% (*v*/*v*) heat-inactivated serum following the protocols recommended by the supplier. Thus, bovine serum was used for preadipocytes while fetal bovine serum was for myoblasts and macrophages (Invitrogen, NZ). Cells were seeded in 12-well plates and maintained in a humidified atmosphere of 5% CO_2_ at 37°C in a standard incubator.

When preadipocytes reached confluence, they were differentiated for 48 hours (h) in complete medium (DMEM containing 25 mM glucose, 10% fetal bovine serum, and antibiotics) and supplemented with dexamethasone (1 mM; Sigma-Aldrich, MO, US), isobutylmethylxantine (0.5 mM; Sigma-Aldrich, MO, US), and insulin (10 *μ*g/ml; Sigma-Aldrich, MO, US). The media were replaced with complete medium and insulin for 48 h. Four days post differentiation cocktail, cell media were replaced with complete medium and changed every 2 days until day 9 post differentiation. On the other hand, myoblasts were differentiated for 48 h with complete medium (DMEM containing 25 mM glucose, 2% horse serum and antibiotics) and supplemented with insulin (10 *μ*g/ml). RAW 264.7 macrophages were grown in complete medium (DMEM containing 25 mM glucose, 10% fetal bovine serum, and antibiotics) until they reached confluence, when they are ready to be treated.

### 2.2. Treatments

Macrophages were activated with LPS (500 ng/ml from *Escherichia coli* K12, InvivoGen, CA, US) for 24 h after cells had reached confluence. To generate a proinflammatory environment in vitro, conditioned medium (CM) was used as previously described [30] to simulate the macrophage infiltration in adipocytes and myotubes for 24 h. This proinflammatory medium was generated using 25% of the medium from activated macrophages with LPS (500 ng/ml for 24 h) and 75% complete medium.

CeO_2_ NPs used for this study (544841; Sigma-Aldrich, MO, US) were previously characterized as reported elsewhere [31]. Nanoparticles were diluted in ultrapure MilliQ water at a concentration of 10 mg/ml. The CeO_2_ NPs were first characterized in terms of size, dispersion, and surface charge. For this purpose, CeO_2_ NPs were diluted in MilliQ water in order to ensure that the light scattering intensity was within the sensitivity range of the instrument. Particle surface charge was determined by Z-potential, based on the study of the surface charge through particle mobility in an electric field. The average particle diameter size and polydispersity index were analyzed by photon correlation spectroscopy. All these data were measured by laser Doppler velocimetry (Zetasizer Nano, Malvern Instruments, UK) using a quartz cell at 25°C with a detection angle of 90°. At least three different batches were analyzed to give an average value and standard deviation for the particle diameter, PDI, and zeta potential. Dilutions to 100 *μ*g/ml, 50 *μ*g/ml, 20 *μ*g/ml, and 10 *μ*g/ml were performed just before the experiments with cell culture medium. The proinflammatory media and CeO_2_ NPs were added simultaneously to cell cultures. The complete medium without proinflammatory conditions (LPS/CM) was used as a control medium. The complete medium without nanoparticles nor proinflammatory stimulation (CM) was used as nontreated control (hereinafter the NTC). The supernatants, intracellular (total cell lysate) proteins, and total RNA were collected with their appropriate reagent and stored at −20°C for subsequent analysis.

### 2.3. Cell Metabolic Assays

The metabolic activity of cells was determined by the 3-(4,5-dimethyl-thiazol-2-yl)-2,5-diphenenyl tetrazolium bromide (MTT; Sigma-Aldrich, MO, US) reduction assay in 96-well plates. The treatments were performed as described in the section above. Cells were incubated for 2 h with 0.45 mg/ml MTT dye to allow the formation of the dark blue formazan crystals generated by living cells. Then, the medium was removed and 100 *μ*l of solubilization solution was added to dissolve the crystals as described in the manufacturer's instructions. Absorbance was read with Multiskan Spectrum (Thermo Scientific, MA, US) at 570/630 nm wavelength.

The effect of the treatment on cellular metabolism was also evaluated through biochemical markers. Thus, glucose uptake (Hk-CP; Horiba, FR), lactate release (A11A01721; ABX Diagnostic, FR), and glycerol release (GLY 105; Randox Laboratories, UK) were measured from supernatants after the 24 h treatment with a PENTRA C200 autoanalyzer (Horiba, FR). Glucose uptake was calculated by the difference between glucose amount (present in the culture media) before and after the incubation period.

Additionally, secreted adiponectin (ADIPOQ), interleukin-6 (IL-6), monocyte chemoattractant pProtein-1 (MCP-1), and tumor necrosis factor alpha (TNF-*α*) were measured in the supernatants using commercial ELISA kits (DY1065, DY406, DY479 and DY410, respectively; R&D, ES). Intracellular levels of the transcription factors hypoxia-inducible factor-1 alpha (HIF-1*α*) were also determined with ELISA kits (DYC1935; R&D, ES), following the manufacturer's instructions. The results were normalized to total protein content as determined by Pierce BCA assay (Thermo Scientific, IL, US).

### 2.4. ROS Production

To determine extra- and intracellular ROS concentration, 2,7-dichlorofluorescein diacetate (DCFH-DA) was used following the guidelines of the supplier. Briefly, cells and supernatants were incubated with 1 *μ*M DCFH-DA for 40 min in a standard incubator (5% CO_2_ at 37°C), then supernatants were loaded on a 96-well plate and fluorescence measured using a POLARstar spectrofluorometer (BMG Labtech, DE) at 485/530 nm. Whereas cells were lysed by freeze-thaw method at -80°C for 2 h and then resuspended in 500 *μ*l phosphate-buffered saline, then the lysates were loaded on a 96-well plate following the same protocol used for supernatants.

### 2.5. Mitochondrial Content

Mitochondria were labelled using MitoTracker Green FM (Molecular Probes, Life Technologies Ltd., Paisley, UK), which reacts with the free thiol groups of cysteine residues belonging to mitochondrial proteins. Cells were incubated with this mitochondria-specific dye according to the manufacturer's protocol at a final concentration of 25 nM for 30 min prior to visualization. For fluorescence intensity quantification, a POLARstar Galaxy spectrofluorometer plate reader (BMG Labtech, DE) was used, set up to 554 nm excitation and 576 nm emission wavelengths. Fluorescent microscopy was performed on living cells with ZOE Fluorescent Cell Imager (Bio-Rad Laboratories, DE).

### 2.6. Analysis of Gene Expression

Total RNA was extracted from treated cells using QIAzol reagent (Qiagen, NL) according to the manufacturer's instructions. A total amount of 2 *μ*g of RNA were transcribed to cDNA using MultiScribe™ MuLV and random primers (High-Capacity cDNA Reverse Transcription Kit; Applied Biosystems, CA, US). Real-time PCR was performed in an ABI Prism 7900HT Fast System Sequence Detection System (Applied Biosystems, CA, US) equipped with the SDS software (version 2.4.1) using SYBR Green (iQ™ SYBR® Green supermix, Bio-Rad Laboratories, DE) and primers designed with Primer-BLAST software (National Center for Biotechnology Information, MD, USA; https://www.ncbi.nlm.nih.gov/tools/primer-blast/), according to published cDNA [30] or genomic sequences (Table S1) and with melting temperatures ranging from 58 to 60°C. A 2-fold dilution series was prepared from pooled cDNA samples to evaluate primer efficiency (*E* = 10^[−1/slope]^) and specificity as described elsewhere [32]. The relative expression was determined by the E^-ΔΔCt^ method after internal normalization to *Ppia* as housekeeping gene.

### 2.7. Statistical Analysis

Data are presented as mean and SEM. Statistical significance and interaction were analyzed by two-way ANOVA followed by Dunnet post hoc test for multiple comparisons when comparing the effect of CeO_2_ NPs at different doses in control or proinflammatory conditions (LPS/CM). One-way ANOVA followed by Dunnet and Kruskal-Wallis test followed by Dunn test for the nonparametric statistics were used to compare the effect of CeO_2_ NPs on gene expression in proinflammatory conditions (LPS/CM). The comparison between the gene expression of two groups (control vs. inflammation) was analyzed by unpaired Student's *t*-test for parametric, and Mann-Whitney *U* test for nonparametric statistics. Statistical analyses and graphs were performed using Prism 5.0 software (GraphPad Software Inc., CA, US). Values of *p* < 0.05 were considered statistically significant.

## 3. Results

### 3.1. Nanoparticle Characterization

Z-potential was measured to analyze the changes on surface charge and, therefore, to estimate the adherence of CeO_2_ NPs to the cells. Negative or positive values are characteristic of stable colloidal systems. However, positive charges might provoke a certain degree of toxicity *in vitro* [33]. Z-potential mean formulation of CeO_2_ NPs was 30.57 ± 0.58 mV. Formulation polydispersity index average was 0.36 ± 0.01. This value is an indicator of the homogeneity of the formulation since nanoparticles with values ranging between 0 and 0.3 are considered acceptable according to dynamic light scattering specifications, while values higher than 0.7 indicate a wide range of distribution. Tested nanoparticles presented a mean size distribution as manufacturer reported (96.7% is ≤25.3 nm in MilliQ water).

### 3.2. Cell Metabolism

**Figure 1:**
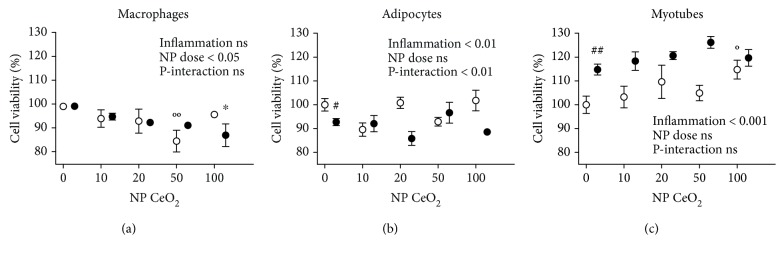
Cell metabolic activity in RAW 264.7 macrophages (a), 3T3-L1 adipocytes (b), and C2C12 myotubes (c) measured with MTT assay at 24 h after CeO_2_ NP treatment at 10, 20, 50, and 100 *μ*g/ml doses in percentage compared to nontreated control (NTC). White shapes: control medium; black shapes: inflammation in macrophages activated with lipopolysaccharide (LPS), adipocytes, and myotubes treated with conditioned medium (CM); ^#^ *p* < 0.05, ^##^ *p* < 0.01 control vs. inflammation; °*p* < 0.05, °°*p* < 0.01 CeO_2_ NPs vs. control; ^∗^ *p* < 0.05 CeO_2_ NPs vs. inflammation; data (*n* = 6/group) are expressed as mean (SEM).

The potential influence of CeO_2_ NPs in cell metabolic activity was tested using MTT assay which mainly measures the cell mitochondrial activity through NAD(P)H-dependent cellular oxidoreductase enzymes.  Figure 1 shows the cell viability of the three different cell types after exposure to CeO_2_ NPs at increasing doses ranging from 10, 20, 50, to 100 *μ*g/ml. First, the inflammatory stimuli (CM vs. NTC) decreased metabolic activity in adipocytes (Figure 1(b)) while increased in myotubes (Figure 1(c)). Moreover, the interaction between the treatment with CeO_2_ NPs and inflammatory status was only statistically significant in adipocytes (Figure 1(b)). The nanoparticles were considered noncytotoxic since the metabolic activity was higher than 80% as compared to each cell type control. However, macrophages showed a statistically significant reduction in the cell metabolic activity at dose 50 *μ*g/ml of CeO_2_ NPs in control medium and 100 *μ*g/ml of CeO_2_ NPs in proinflammatory conditions (LPS) (Figure 1(a)). Conversely, the effect on myotubes was the opposite, increasing the metabolism at the dose of 100 *μ*g/ml CeO_2_ NPs in control medium (Figure 1(c)).

To further analyze if the CeO_2_ NP treatment affects cellular metabolism, glucose uptake, lactate release, and glycerol release were determined in supernatants after 24 h of CeO_2_ NP treatment. Inflammation (LPS/CM vs. NTC) increased glucose uptake in all cell types analyzed (Figure S1(a-c)), while lactate release and glycerol release were higher only in macrophages and adipocytes, respectively (Figure S1(d, g)). There was no significant interaction between the treatment with CeO_2_ NPs and inflammatory status in glucose uptake, lactate release, and glycerol release in any cell type. Glucose uptake and lactate release showed a statistically significant increase in macrophages in proinflammatory conditions (LPS) treated with 10 and 50 *μ*g/ml CeO_2_ NPs, respectively (Figure S1(a, d)). Beyond that, neither macrophages, adipocytes, nor myotubes showed an alteration in the levels of the metabolic markers determined. In addition, anaerobic metabolism (calculated through the lactate generated over glucose consumption) remained unchanged in all cell types (data not shown).

Moreover, to test the effect of CeO_2_ NPs on inflammation, the secretion of several metabolic-related cytokines was measured in supernatants after 24 h of treatment. The secretion of IL-6, MCP-1, and TNF-*α* was increased in both macrophages (Figure S2(a, d, g)) and adipocytes (Figure S2(b, e, h)) in proinflammatory conditions (LPS/CM vs. NTC). IL-6 and TNF-*α* release was induced in myotubes in proinflammatory conditions (CM vs. NTC) (Figure S2(c, i)). Moreover, ADIPOQ secretion was lower in adipocytes and myotubes under proinflammatory conditions (CM vs. NTC) (Figure S2(j, k)). An interaction effect between the treatment inflammation was found in the secretion of IL-6 in myotubes (Figure S2(c)), as well as in TNF-*α* in macrophages (Figure S2(g)). The treatment with CeO_2_ NPs in control medium does not affect the release of the cytokines selected as metabolic-related markers in any cell type. On the other hand, a statistically significant increase of IL-6 was observed in myotubes under proinflammatory conditions (CM) at dose 10 and 50 *μ*g/ml of CeO_2_ NPs (Figure S2(c)). MCP-1 levels were lower in macrophages under proinflammatory conditions (LPS) at dose 20 *μ*g/ml of CeO_2_ NPs (Figure S2(d)). TNF-*α* increased in macrophages at dose 20 and 50 *μ*g/ml of CeO_2_ NPs (Figure S2(g)). ADIPOQ release did not change in adipocytes and myotubes under proinflammatory conditions (CM) after the CeO_2_ NP treatment (Figure S2(j, k)). Furthermore, HIF-1*α* was measured to explore the potential effects of CeO_2_ NPs on inflammation-derived activation of this master regulator of the hypoxic cascade. The results showed a lack of effect of these CeO_2_ NPs concerning the hypoxic cascade in both adipocytes (Figure S3(b)) and myotubes (Figure S3(c)). However, macrophages under proinflammatory conditions (LPS vs. NTC) increased the levels of HIF-1*α* after the treatment with CeO_2_ NPs at dose 10 *μ*g/ml while decreased at dose 50 *μ*g/ml (Figure S3(a)).

### 3.3. Antioxidant Activity

**Figure 2:**
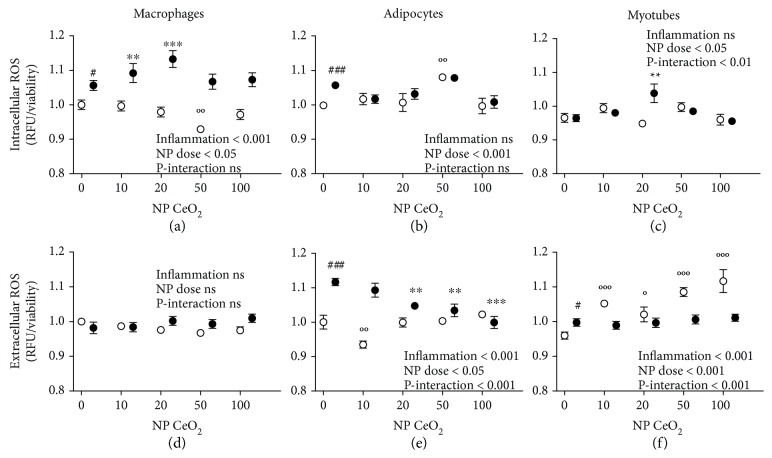
Intra- and extracellular ROS production in RAW 264.7 macrophages (a, d), 3T3-L1 adipocytes (b, e), and C2C12 myotubes (c, f) measured with DCFH-DA assay at 24 h after CeO_2_ NP treatment at 10, 20, 50, and 100 *μ*g/ml doses in percentage compared to nontreated control (NTC). White shapes: control medium; black shapes: inflammation in macrophages activated with lipopolysaccharide (LPS), adipocytes, and myotubes treated with conditioned medium (CM); ^#^ *p* < 0.05, ^###^ *p* < 0.001 control vs. inflammation; °*p* < 0.05, °°*p* < 0.01, °°°*p* < 0.001 CeO_2_ NPs vs. control; ^∗∗^ *p* < 0.01, ^∗∗∗^ *p* < 0.001 CeO_2_ NPs vs. inflammation; data (*n* = 6/group) are expressed as mean (SEM).

Intra- and extracellular antioxidant activity of CeO_2_ NPs was evaluated with the fluorophore DCFH-DA. Inflammation (LPS/CM vs. NTC) increased intracellular ROS levels in macrophages and adipocytes (Figures 2(a) and 2(b)), as well as induced extracellular ROS production in adipocytes and myotubes (Figures 2(e) and 2(f)). An interaction effect was detected between the treatment with CeO_2_ NPs and inflammatory status in the intracellular ROS production in myotubes (Figure 2(c)), as well as in the extracellular ROS levels in adipocytes and myotubes (Figures 2(e) and 2(f)). Intracellular ROS levels were significantly increased in macrophages and myotubes (Figures 2(a) and 2(c)) at a dose 20 *μ*g/ml of CeO_2_ NPs with inflammation (LPS and CM, respectively) and in adipocytes at 50 *μ*g/ml of CeO_2_ NPs in control medium (Figure 2(b)). Furthermore, a statistically significant reduction was found on intracellular ROS in macrophages at a dose 50 *μ*g/ml of CeO_2_ NPs in control medium (Figure 2(a)). On the other hand, the extracellular ROS levels were reduced in adipocytes at 20, 50, and 100 *μ*g/ml of CeO_2_ NPs in proinflammatory conditions (CM), thus in a dose-dependent manner, as well as at dose 10 *μ*g/ml of CeO_2_ NPs in control medium (Figure 2(e)). Finally, ROS levels were increased extracellularly in myotubes at any dose of CeO_2_ NPs with control medium (Figure 2(f)). No statistically significant scavenging effects of CeO_2_ NPs were seen (intra- and extracellularly) in macrophages (Figures 2(a) and 2(d)) either in control medium or proinflammatory conditions (LPS).

### 3.4. Mitochondria Quantification

**Figure 3:**
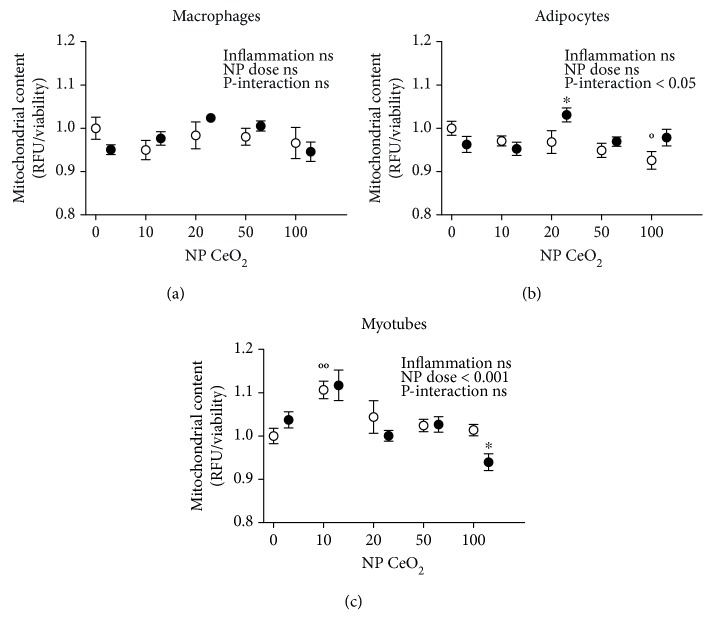
Mitochondrial content in RAW 264.7 macrophages (a), 3T3-L1 adipocytes (b), and C2C12 myotubes (c) analyzed with MitoTracker Green assay at 24 h after CeO_2_ NP treatment at 10, 20, 50, and 100 *μ*g/ml doses in fold change compared to nontreated control (NTC). White shapes: control medium; black shapes: inflammation in macrophages activated with lipopolysaccharide (LPS), adipocytes, and myotubes treated with conditioned medium (CM); °*p* < 0.05, °°*p* < 0.01 CeO_2_ NPs vs. control; ^∗^ *p* < 0.05 CeO_2_ NPs vs. inflammation; data (*n* = 6/group) are expressed as mean (SEM).

To assess the potential effects of CeO_2_ NPs on mitochondrial content, MitoTracker Green fluorescent probe was used. No statistically significant effects on mitochondrial quantification were found when comparing the proinflammatory conditions with NTC in any of the cell types (Figure 3). Additionally, adipocytes showed a treatment-inflammation interaction in mitochondria number (Figure 3(b)). A statistically significant increase in the mitochondrial content was observed in both adipocytes at 20 *μ*g/ml of CeO_2_ NPs in proinflammatory conditions (CM) and myotubes at 10 *μ*g/ml of CeO_2_ NPs in control medium, while a decrease was detected in adipocytes at dose 100 *μ*g/ml of CeO_2_ NPs in control medium and myotubes at the same dose but in proinflammatory conditions (CM) (Figures 3(b) and 3(c)).

### 3.5. Gene Expression Patterns

**Figure 4:**
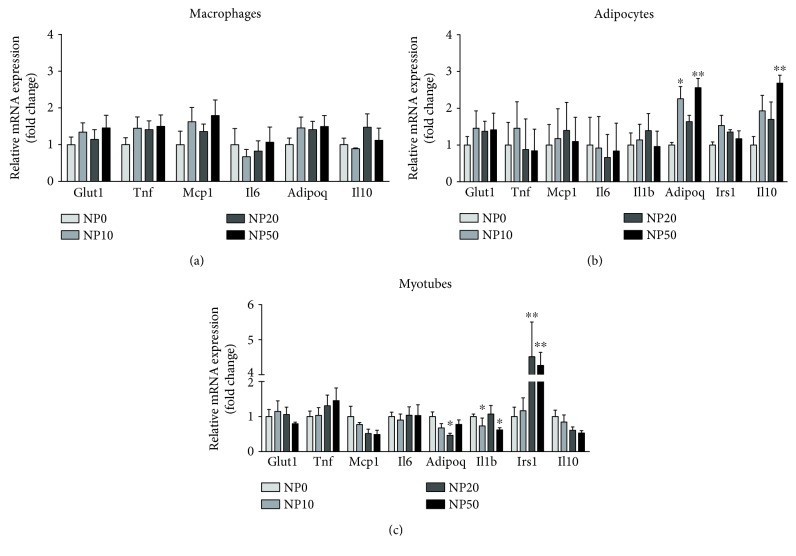
Relative mRNA expression levels in RAW 264.7 macrophages with LPS (a), 3T3-L1 adipocytes with CM (b), and C2C12 myotubes with CM (c) at 24 h after CeO_2_ NP treatment at 10, 20, and 50 *μ*g/ml doses. Normalized to *Ppia* housekeeping gene in fold change compared to nontreated control (NTC). ^∗^ *p* < 0.05, ^∗∗^ *p* < 0.01; data (*n* = 6/group) are expressed as mean (SEM). CM: conditioned medium; LPS: lipopolysaccharide.

The most representative genes for metabolism-related comorbidities that changed at least in one cell type under proinflammatory stimuli compared to their controls were further analyzed by real-time PCR (Figure S4). To determine whether CeO_2_ NPs could attenuate the proinflammatory stimulation (LPS/CM), the expression of candidate genes was measured as a screening of the pathways that potentially could be involved in the effects observed in the assays. No statistically significant differences were found in mRNA expression of selected genes in macrophages incubated with CeO_2_ NPs (Figure 4(a)). The expression of *Adipoq* significantly increased in adipocytes at doses of 10 and 50 *μ*g/ml of CeO_2_ NPs and *Il10* at 50 *μ*g/ml (Figure 4(b)). Furthermore, in myotubes, both *Il1b* at 20 *μ*g/ml and *Adipoq* at 10 and 50 *μ*g/ml of CeO_2_ NPs were downregulated, while *Irs1* showed a statistically significant increase at 20 and 50 *μ*g/ml of CeO_2_ NPs (Figure 4(c)).

## 4. Discussion

In this study, we have shown that murine macrophages, adipocytes, and myotubes treated with CeO_2_ NPs could improve insulin sensitivity-related features at cellular level, after being exposed to proinflammatory stimuli. This research suggests that an in vitro treatment with CeO_2_ NPs (without inflammatory stimuli) does not clearly improve the response of the oxidative and inflammatory pathways. On the other hand, a potential effect on insulin resistance was found in metabolic syndrome-related cell lines (myotubes and adipocytes) under proinflammatory stimuli by means of modulating the oxidative status, mitochondrial content, and gene expression. The effect of some insulin-sensitizing molecules could be related to the increased mitochondrial content, as type 2 diabetes features are related to lower mitochondria presence [7].

Oxidative stress and inflammation activate the gene transcription of many inflammatory factors, some of them are subsequently translated into secreted cytokines, which are proteins that are released and act to nearby (paracrine) or distant (endocrine) cells. The increased levels of proinflammatory cytokines (TNF-*α*, IL-6, and IL-1*β*) have been found to be important contributors to the underlying processes of the development of metabolic syndrome [5]. Although the implication of IL-6 has tended to be on proinflammatory signaling activation, recent studies suggested a dual role in the homeostatic control of metabolism, for instance, mice lacking *Il6* gene develop insulin resistance and liver inflammation, while patients receiving IL-6R blocking drug therapy increased body weight and developed dyslipidemia [34]. Moreover, the skeletal muscle-derived IL-6 has been suggested to have beneficial effects, modulating glucose and fatty acid metabolism during exercise but also contributing to the development of insulin resistance when chronically elevated [35]. In our experiments, CeO_2_ NP treatment increased IL-6 release in myotubes under proinflammatory conditions, which could influence insulin sensitivity. However, no significant differences were observed in metabolic markers (glucose, lactate, and glycerol) in any of the cell types assayed in our study but an increase in glucose uptake and lactate release in macrophages under proinflammatory conditions treated with CeO_2_ NPs, which suggests that the potential benefit on insulin resistance upon CeO_2_ NP treatment might rely on other (*in vivo*) mechanisms which could not be considered in our experimental setting.

The research on the beneficial effects of nanoceria is still inconclusive, as several studies obtained contradictory findings about their biological activity. Several authors reported anti-inflammatory and antioxidant properties of CeO_2_ NPs on cell cultures of murine macrophages [17], cardiomyocytes [23], mesenchymal stem cells, and *β*-cells [36] as well as neuronal-like cells [31]. *In vivo* animal studies showed beneficial effects of CeO_2_ NPs on preventing weight gain accompanied by a decrease in plasma insulin, leptin, glucose, and triglycerides [25], reducing retinal neurodegenerative disease [19] and cardiac dysfunction [22], attenuating hypoxia-derived lung damage [20], and alleviating liver ROS toxicity [21] among others. Conversely, other studies evidenced a lack of effectiveness on human monocytes [27, 37] or even deleterious effects on this cell type [29] and oxidative stress and inflammation in the lung, liver, kidney, heart, spleen, and brain of mice [26]. Moreover, these nanoparticles were used to induce cytotoxicity and oxidative damage in tumor cells [15, 28] at the same time protecting nonmalignant cells from chemotherapy [15]. The differences in biological targets (cell types and species), experimental designs (exposure to inflammation/oxidants for treatment or with the nanoparticles for prevention), nanoparticles (synthesis method, size, shape, and chemical characteristics), and objectives of the studies could lead to these variations, being the outcome interpretation and comparison highly complex. The dose of CeO_2_ NPs used in the present study has been selected from previous studies involving 3T3-L1 adipocytes and rat mesenchymal stem cells which assessed the impact of these nanoparticles on adipogenesis and obesity-related parameters in rodents [24, 25]. As reported in our experimental assay, none of the doses used in this study seem to induce cell damage regarding to MTT assay data. However, the higher concentration of CeO_2_ NPs (100 *μ*g/ml) decreased the mitochondrial content and increased extracellular ROS levels in myotubes, and therefore it was not analyzed in functional assays.

The beneficial effect of nanoparticles in cell cultures could differ due to diverse biochemical characteristics, for instance a lower pH could drive them to act as oxidants and thus generating ROS [24]. The relative proportion of charges varies with the different methods used to prepare the nanoparticles [13]. These findings are of particular interest as the surface oxidation state of the CeO_2_ NPs has been demonstrated to alter its enzyme-mimetic activity, thereby the ability of the nanoparticles to scavenge superoxide is directly related to Ce^+3^ concentrations at its surface [38]. In this sense, lower Ce^+3^/Ce^+4^ ratios were found to be less efficient [16].

The novelty of the present findings is that CeO_2_ NPs were tested in cell cultures under proinflammatory conditions, which are likely to be present in the event of therapeutic application of CeO_2_ NPs in metabolic syndrome-related organs, thus representing a more physiological approach for evaluating their therapeutic properties [30]. Besides the oxidative stress pathways, we also tested the protective effect of the nanoparticles on the inflammatory response albeit with inconclusive results. The interactions found in the present study between inflammation and the treatment with CeO_2_ NPs in a large number of assays evidenced the differential effects of this potential therapy depending on the inflammatory status. Indeed, some authors recommended the evaluation of the nanomaterial therapeutic potential in the presence of immunomodulators [27], similar to the use of LPS and CM as proinflammatory stimuli in the present work. On the other hand, we found little beneficial effect of CeO_2_ NPs on lipopolysaccharide-induced cytokine release from macrophages, suggesting that the previously reported effects in this cell type may be limited in their scope of action and do not extend to a general downregulation of the inflammatory response. Furthermore, we found a reduction in the viability of macrophages that could be explained by the lower cytoplasmic volume where the nanoparticles could be more concentrated and thus more toxic as previously described [37].

## 5. Conclusion

Overall, our results suggest that CeO_2_ NPs could have a potential insulin-sensitizing effect specifically on adipose tissue and skeletal muscle as related to mitochondrial function. Nevertheless, the treatment does not seem to alter, in a physiologically relevant manner, the response of the oxidative and inflammatory pathways. Our results emphasize the need to evaluate the effects of nanoparticles in the presence of stimulators (LPS or CM) which are expected to occur *in vivo* under metabolic syndrome and its related conditions. Additional studies on primary human cells focusing on susceptible populations (with preexisting diseases), investigating the time, dose, and mechanism of action are necessary for the identification of the real benefits and hazards of CeO_2_ NPs.

## Data Availability

The data used to support the findings of this study are available from the corresponding author upon request.

## Supplementary Materials

Supplementary Materials Table S1: primer sequences for the quantitative PCR for the mouse genes analyzed. The gene identification number (ID) is the unique identifier number from the Entrez Global Query Cross-Database Search System at the National Center for Biotechnology Information. Figure S1: glucose uptake, lactate release, and glycerol release in RAW 264.7 macrophages (a, d), 3T3-L1 adipocytes (b, e, g), and C2C12 myotubes (c, f) at 24 h after CeO_2_ NP treatment at 10, 20, and 50 *μ*g/ml doses in fold change compared to nontreated control (NTC). White shapes: control medium; black shapes: inflammation in macrophages activated with lipopolysaccharide (LPS), adipocytes, and myotubes treated with conditioned medium (CM); ^#^ *p* < 0.05, ^###^ *p* < 0.001 control vs. inflammation; ^∗∗^ *p* < 0.01 CeO_2_ NPs vs. inflammation; data (*n* = 6/group) are expressed as mean (SEM). Figure S2: secretion of IL-6, MCP-1, TNF-*α*, and ADIPOQ in RAW 264.7 macrophages (a, d, g), 3T3-L1 adipocytes (b, e, h, j), and C2C12 myotubes (c, f, i, k) at 24 h after CeO_2_ NP treatment at 10, 20, and 50 *μ*g/ml doses in *ρ*g/mg total protein compared to nontreated control (NTC). C: control medium; LPS or CM: inflammation in macrophages activated with lipopolysaccharide (LPS), adipocytes, and myotubes treated with conditioned medium (CM); ^##^ *p* < 0.01, ^###^ *p* < 0.001 control vs. inflammation; ^∗^ *p* < 0.05, ^∗∗^ *p* < 0.01, ^∗∗^ *p* < 0.001 CeO_2_ NPs vs. inflammation; data (*n* = 6/group) are expressed as mean (SEM). Figure S3: HIF-1*α* total protein in RAW 264.7 macrophages (a), 3T3-L1 adipocytes (b), and C2C12 myotubes (c) at 24 h after CeO_2_ NP treatment at 10, 20, and 50 *μ*g/ml doses in *ρ*g/total protein compared to nontreated control (NTC). C: control medium; LPS or CM: inflammation in macrophages activated with lipopolysaccharide (LPS), adipocytes, and myotubes treated with conditioned medium (CM); ^#^ *p* < 0.05 control vs. inflammation; ^∗^ *p* < 0.05 NPs vs. inflammation; data (*n* = 6/group) are expressed as mean (SEM). Figure S4: relative mRNA analysis of metabolism-related markers in RAW 264.7 macrophages activated with LPS (a), 3T3-L1 adipocytes (b), and C2C12 myocytes (c) treated with conditioned medium (CM). Results normalized to Ppia housekeeping gene. ^∗^ *p* < 0.05, ^∗∗^ *p* < 0.01, ^∗∗∗^ *p* < 0.001 control vs. inflammation (LPS or CM); data (*n* = 6/group) are expressed as mean (SEM).
