# Supplementary material for: Cerium Oxide Nanoparticles Regulate Insulin Sensitivity and Oxidative Markers in 3T3-L1 Adipocytes and C2C12 Myotubes
Source: Oxid Med Cell Longev. 2019 Feb 4;2019:2695289. doi: 10.1155/2019/2695289 (PMC6378795; doi:10.1155/2019/2695289)
Supplement: Supplementary Materials — Table S1: primer sequences for the quantitative PCR for the mouse genes analyzed. The gene identification number (ID) is the unique identifier number from the Entrez Global Query Cross-Database Search System at the National Center for Biotechnology Information. Figure S1: glucose uptake, lactate release, and glycerol release in RAW 264.7 macrophages (a, d), 3T3-L1 adipocytes (b, e, g), and C2C12 myotubes (c, f) at 24 h after CeO2 NP treatment at 10, 20, and 50 μg/ml doses in fold change compared to nontreated control (NTC). White shapes: control medium; black shapes: inflammation in macrophages activated with lipopolysaccharide (LPS), adipocytes, and myotubes treated with conditioned medium (CM); # p < 0.05, ### p < 0.001 control vs. inflammation; ∗∗ p < 0.01 CeO2 NPs vs. inflammation; data (n = 6/group) are expressed as mean (SEM). Figure S2: secretion of IL-6, MCP-1, TNF-α, and ADIPOQ in RAW 264.7 macrophages (a, d, g), 3T3-L1 adipocytes (b, e, h, j), and C2C12 myotubes (c, f, i, k) at 24 h after CeO2 NP treatment at 10, 20, and 50 μg/ml doses in ρg/mg total protein compared to nontreated control (NTC). C: control medium; LPS or CM: inflammation in macrophages activated with lipopolysaccharide (LPS), adipocytes, and myotubes treated with conditioned medium (CM); ## p < 0.01, ### p < 0.001 control vs. inflammation; ∗ p < 0.05, ∗∗ p < 0.01, ∗∗ p < 0.001 CeO2 NPs vs. inflammation; data (n = 6/group) are expressed as mean (SEM). Figure S3: HIF-1α total protein in RAW 264.7 macrophages (a), 3T3-L1 adipocytes (b), and C2C12 myotubes (c) at 24 h after CeO2 NP treatment at 10, 20, and 50 μg/ml doses in ρg/total protein compared to nontreated control (NTC). C: control medium; LPS or CM: inflammation in macrophages activated with lipopolysaccharide (LPS), adipocytes, and myotubes treated with conditioned medium (CM); # p < 0.05 control vs. inflammation; ∗ p < 0.05 NPs vs. inflammation; data (n = 6/group) are expressed as mean (SEM). Figure S4: relative mRNA analysis of meta [file 2695289.f1.pdf]

Supplementary Material

TABLE S1. Primer sequences for the quantitative PCR for the mouse genes analyzed. The gene identification number (ID) is the unique identifier number from the Entrez Global Query Cross-Database Search System at the National Center for Biotechnology Information.

Gene symbol	Gene name	Entrez Gene ID		Sequence (5'→3')	Product Size (bp)
Adipoq	adiponectin, C1Q and collagen domain containing	11450	F:<br>R:	gtcagtggatctgacgacaccaa<br>atgcctgccatccaacctg	171bp
Glut1	solute carrier family 2 member 1	20525	F:<br>R:	cttcattgtgggcatgtgcttc<br>aggttcggcctttggtctcag	134bp
Il1b	interleukin 1 beta	16176	F:<br>R:	gcaactgttcctgaactcaact<br>atcttttggggtccgtcaact	89bp
Il6	interleukin 6	16193	F:<br>R:	ccacttcacaagtcggaggctta<br>gcaagtgcatcatcgttgttcatac	112bp
Il10	interleukin 10	16153	F:<br>R:	catggcccagaaatcaagga<br>ggagaaatcgatgacagcgc	91bp
Irs1	insulin receptor substrate 1	16367	F:<br>R:	gcgggctgactccaagaac<br>gctatccgcggcaatgg	76bp
Mcp1	chemokine (C-C motif) ligand 2	20296	F:<br>R:	ttaacgccccactcacctgctg<br>gcttctttgggacacctgctgc	106bp
Ppia	peptidylprolyl isomerase A	268373	F:<br>R:	ctgagcactggggagaaagga<br>gaagtcaccaccctggcaca	87bp
Tnf	tumor necrosis factor	21926	F:<br>R:	catcttctcaaaattcgagtgacaa<br>tgggagtagacaaggtacaaccc	175bp

F: Forward; R: Reverse; Bp: base pair.

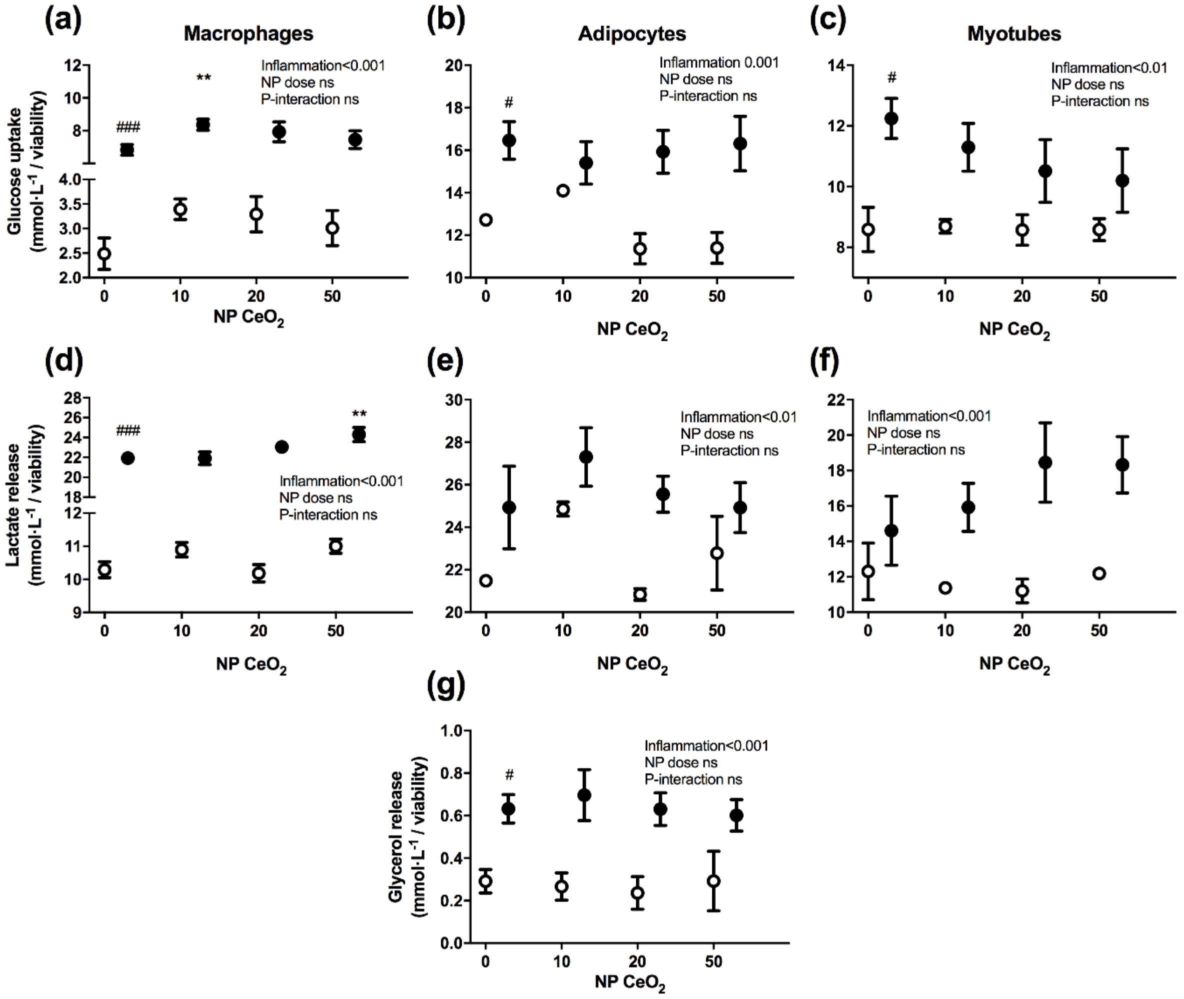

FIGURE S1. Glucose uptake, lactate and glycerol release in RAW 264.7 macrophages (a,d), 3T3-L1 adipocytes (b,e,g) and C2C12 myotubes (c,f) at 24 h after CeO_2 NPs treatment at 10, 20 and 50 µg/ml doses in fold change compared to non-treated control (NTC). White shapes: Control medium; Black shapes: Inflammation in macrophages activated with Lipopolysaccharide (LPS), adipocytes and myotubes treated with conditioned medium (CM); # $p<0.05$, ### $p<0.001$ Control vs. Inflammation; ** $p<0.01$ CeO_2 NPs vs. Inflammation; Data (n=6 /group) are expressed as mean (SEM).

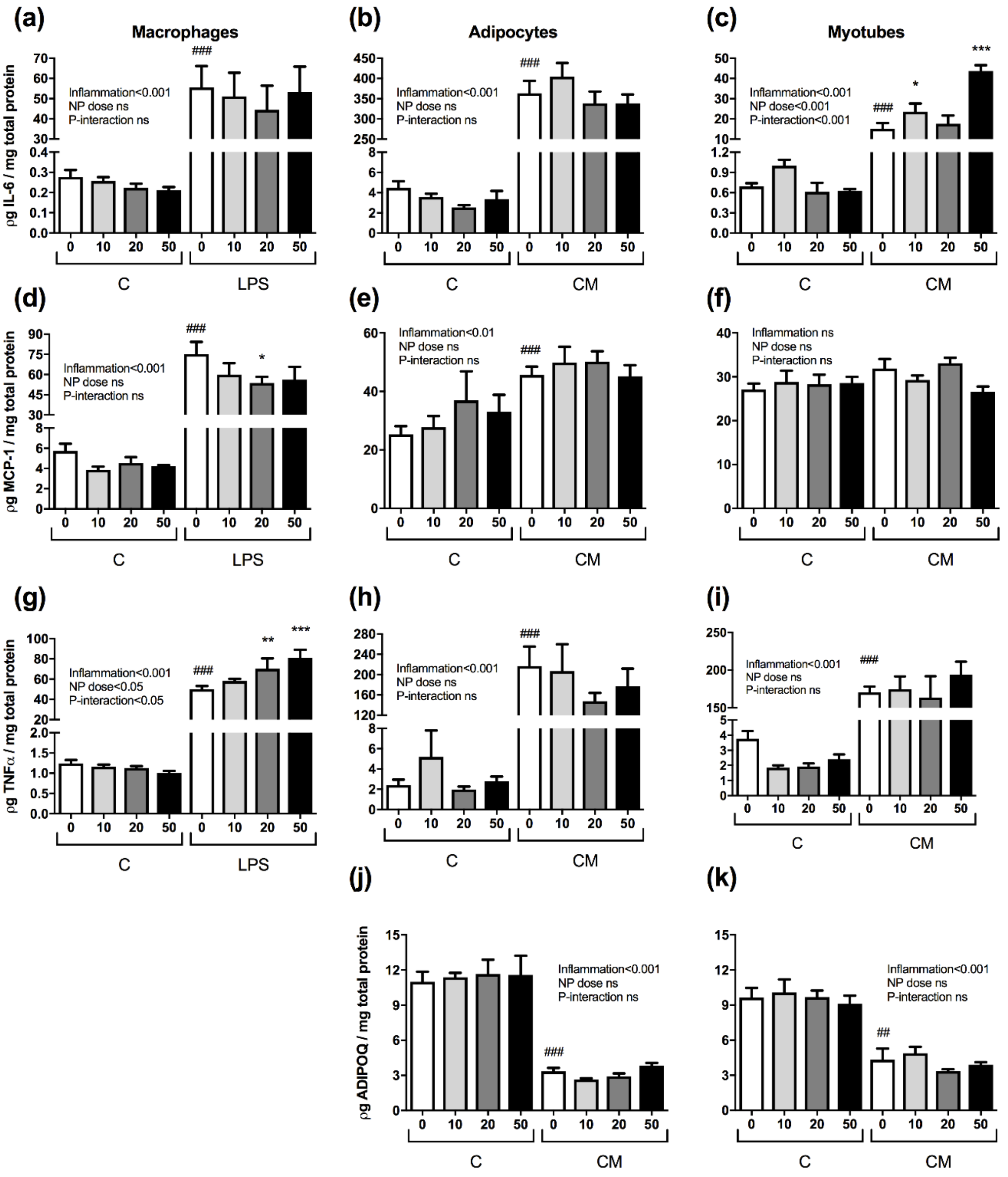

FIGURE S2. Secretion of IL-6, MCP-1, TNF-α and ADIPOQ in RAW 264.7 macrophages (a,d,g), 3T3-L1 adipocytes (b,e,h,j) and C2C12 myotubes (c,f,i,k) at 24 h after CeO_2 NPs treatment at 10, 20 and 50 µg/ml doses in ρg/mg total protein compared to non-treated control (NP0). C: Control medium; LPS or CM: Inflammation in macrophages activated with Lipopolysaccharide (LPS), adipocytes and myotubes treated with conditioned medium (CM); ## p<0.01, ### p<0.001 Control vs. Inflammation; * p<0.05, ** p<0.01, ** p<0.001 CeO_2 NPs vs. Inflammation; Data (n=6 /group) are expressed as mean (SEM).

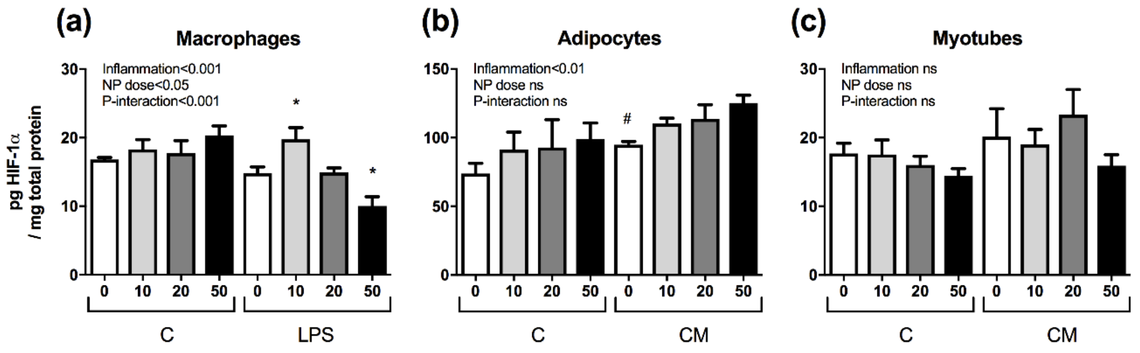

FIGURE S3. HIF-1α total protein in RAW 264.7 macrophages (a), 3T3-L1 adipocytes (b) and C2C12 myotubes (c) at 24 h after CeO_2 NPs treatment at 10, 20 and 50 μg/ml doses in ρg/total protein compared to non-treated control (NTC). C: Control medium; LPS or CM: Inflammation in macrophages activated with Lipopolysaccharide (LPS), adipocytes and myotubes treated with conditioned medium (CM); # $p<0.05$ Control vs. Inflammation; * $p<0.05$ NPs vs. Inflammation; Data (n=6 /group) are expressed as mean (SEM).

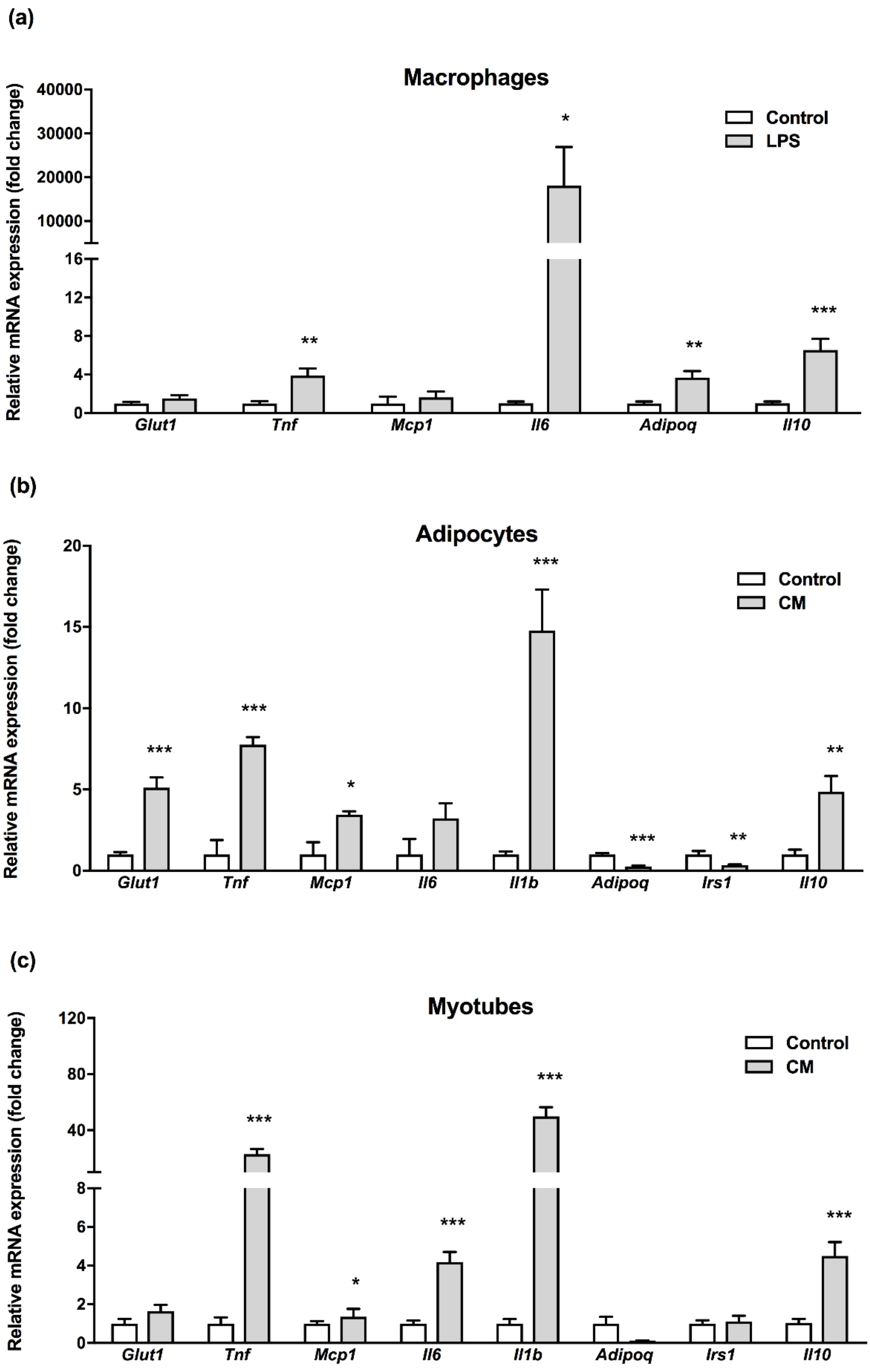

FIGURE S4. Relative mRNA analysis of metabolism-related markers in RAW 264.7 macrophages activated with LPS (a), 3T3-L1 adipocytes (b) and C2C12 myocytes (c) treated with conditioned medium (CM). Results normalized to *Ppia* housekeeping gene. * $p<0.05$, ** $p<0.01$, *** $p<0.001$ Control vs. Inflammation (LPS or CM); Data (n=6 /group) are expressed as mean (SEM).
